# HyperMRI: hyperspectral and magnetic resonance fusion methodology for neurosurgery applications

**DOI:** 10.1007/s11548-024-03102-5

**Published:** 2024-05-18

**Authors:** Manuel Villa, Jaime Sancho, Gonzalo Rosa, Miguel Chavarrias, Eduardo Juarez, Cesar Sanz

**Affiliations:** CITSEM, Universidad Politécnica de Madrid, 28031 Madrid, Spain

**Keywords:** Hyperspectral, Image registration, MRI, Computer-assisted intervention

## Abstract

**Purpose:**

Magnetic resonance imaging (MRI) is a common technique in image-guided neurosurgery (IGN). Recent research explores the integration of methods like ultrasound and tomography, among others, with hyperspectral (HS) imaging gaining attention due to its non-invasive real-time tissue classification capabilities. The main challenge is the registration process, often requiring manual intervention. This work introduces an automatic, markerless method for aligning HS images with MRI.

**Methods:**

This work presents a multimodal system that combines RGB-Depth (RGBD) and HS cameras. The RGBD camera captures the patient’s facial geometry, which is used for registration with the preoperative MR through ICP. Once MR-depth registration is complete, the integration of HS data is achieved using a calibrated homography transformation. The incorporation of external tracking with a novel calibration method allows camera mobility from the registration position to the craniotomy area. This methodology streamlines the fusion of RGBD, HS and MR images within the craniotomy area.

**Results:**

Using the described system and an anthropomorphic phantom head, the system has been characterised by registering the patient’s face in 25 positions and 5 positions resulted in a fiducial registration error of 1.88 ± 0.19 mm and a target registration error of 4.07 ± 1.28 mm, respectively.

**Conclusions:**

This work proposes a new methodology to automatically register MR and HS information with a sufficient accuracy. It can support the neurosurgeons to guide the diagnosis using multimodal data over an augmented reality representation. However, in its preliminary prototype stage, this system exhibits significant promise, driven by its cost-effectiveness and user-friendly design.

## Introduction

Magnetic resonance imaging (MRI) has become one of the most important techniques in the image-guided neurosurgery (IGN) field. This technology allows the generation of multi-planar images of the human body’s internal structures without posing any contraindication, enabling differentiation among various types of tissues. For this reason, MRI has proven its effectiveness in neurosurgery for several purposes. It includes clinical diagnosis [13], treatment planning [22], or surgery assistance [16]. However, despite the evident advantages of using MRI, this solution presents some limitations.

**Fig. 1:**
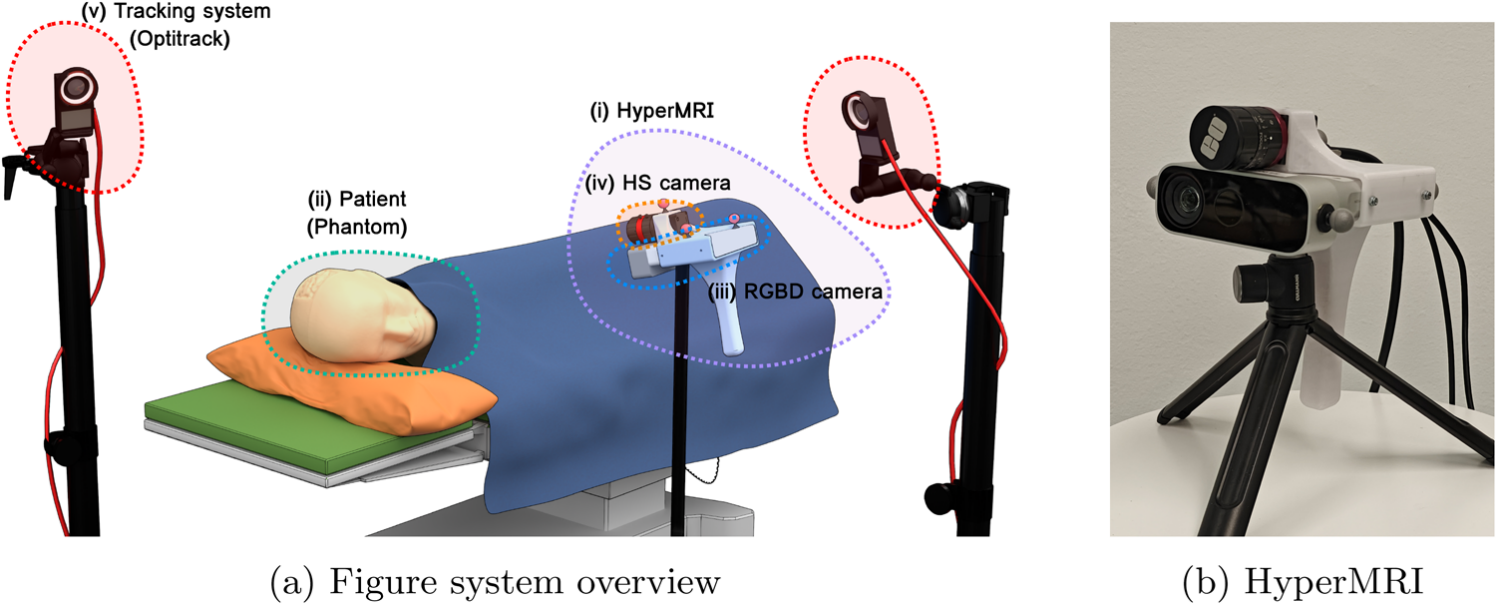
HyperMRI system

One fundamental problem is that the diagnosis relies on the neurosurgeon’s criteria when interpreting the results, requiring extensive prior knowledge and being susceptible to errors. For this reason, many researchers have addressed the task of automatizing the MRI interpretation by means of machine learning (ML) algorithms [6, 15, 19], in some cases mixing the magnetic resonance (MR) information with other medical image sources such as computed tomography (CT), positron emission tomography (PET) [20], ultrasounds (US) [8], or hyperspectral (HS) imaging (HSI) [9, 14]. Another key challenge is the visualization, as the volumetric information generated by the MR is not easily interpretable on a 2D screen. This has initiated a research line on augmented reality (AR) [2, 11] to enhance the human–computer interaction (HCI).

This work aims to address the previous limitations by proposing HyperMRI, a novel AR multimodal methodology and system based on MRI and HSI for assisting brain tumour resection operations. To do so, we introduce a markerless, costless and scalable method to perform a registration between a group of rigidly attached cameras and the MR 3D model. In this way, the HS real-time information captured during the surgical information can be mixed with the MR brain model information. The proposed methodology is devised to be employed under an external tracking system, allowing to preserve the registration even if the camera group moves. The use of an external tracking system also allows a seamless integration of HyperMRI into common neuronavigation systems, as they are based on the same tracking technology [1].

**Fig. 2:**
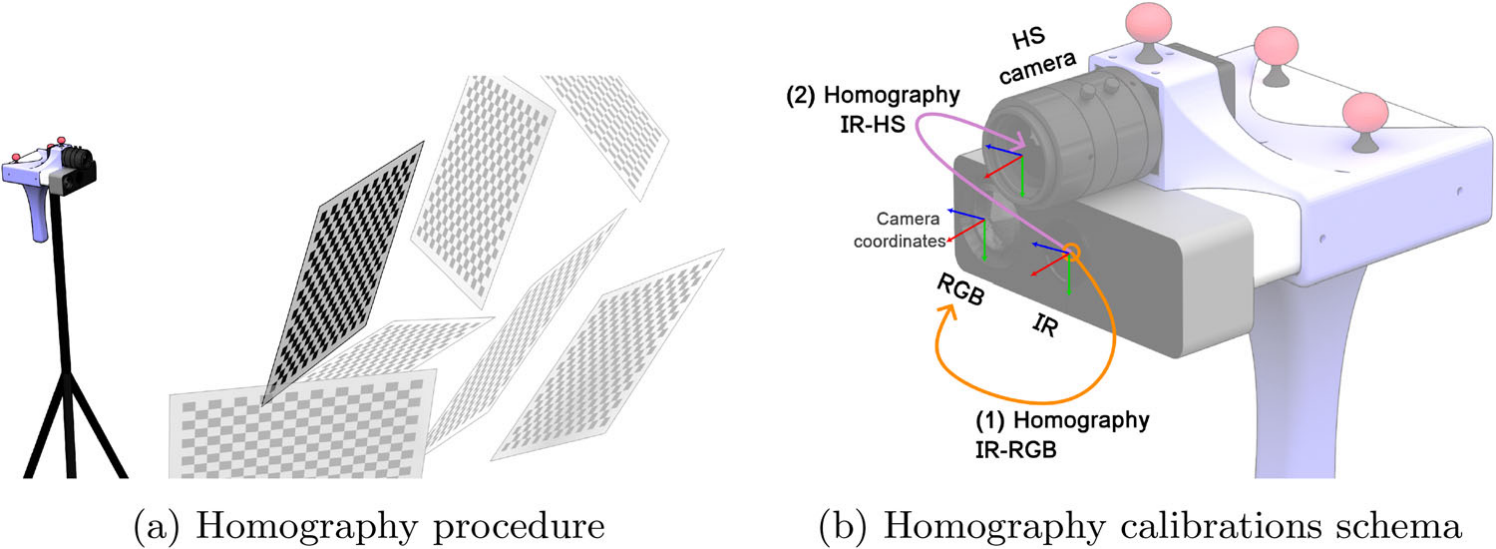
Homography calibration

## Materials and methods

HyperMRI aims to work under the scenario presented in Fig. 1a. In this scenario, (i) HyperMRI is employed to register the preoperative MR information with the actual patient. (ii) HyperMRI (detailed in Fig. 1b) is composed of a rigidly attached Azure Kinect DK RGBD camera (Microsoft) that provides essential geometric data through a depth-sensing device (iii) and HS snapshot camera (Ximea) (iv). The HS camera is employed to perform a tissue classification. The rigid group of cameras also features infrared markers to allow system tracking using an external tracking system (v). This way, the camera can freely move in the space without losing the registration.

**Fig. 3:**
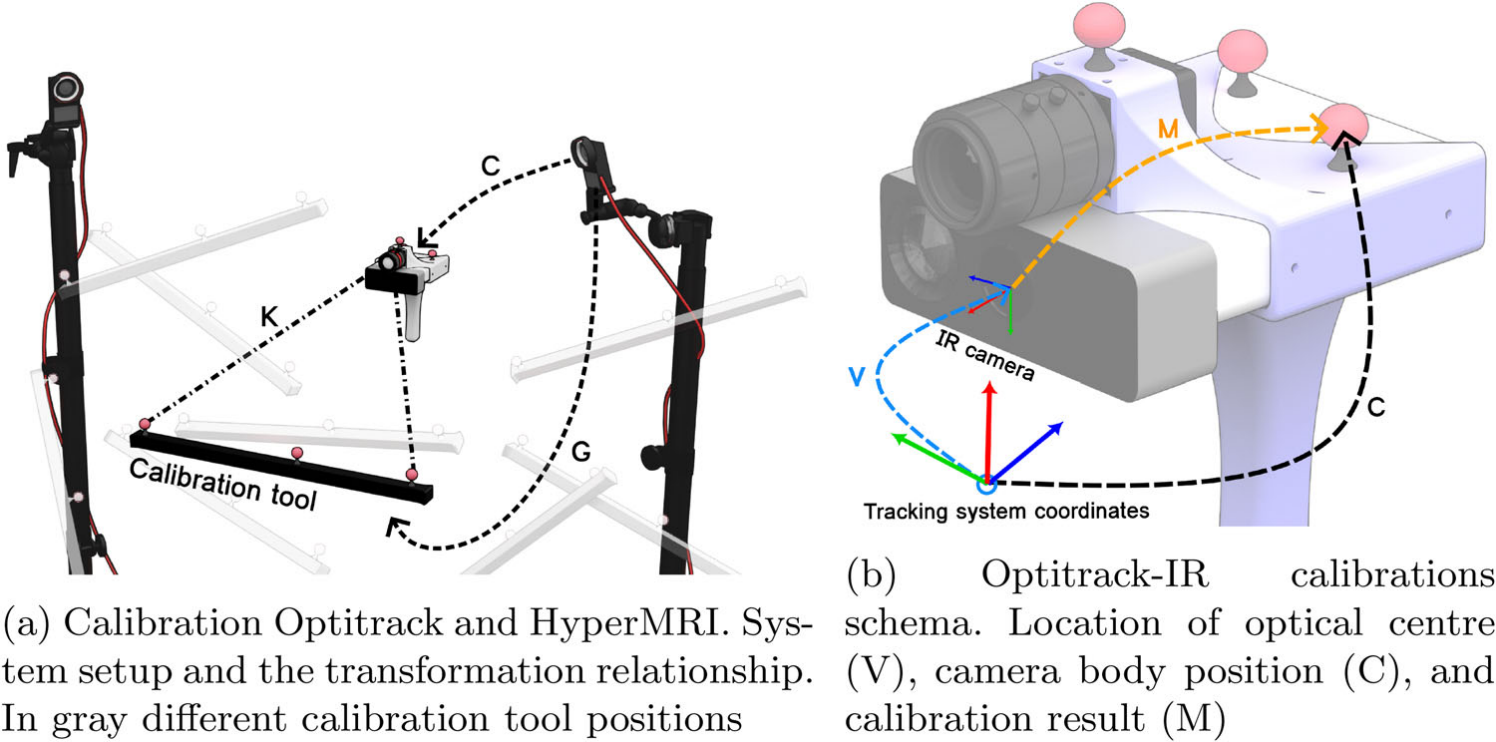
Optitrack-IR calibration

To ensure the precise integration of the HS camera with the MR, in this work, two critical calibration procedures are implemented. First, a homography calibration is conducted to establish the correct information projection from the HS camera onto the RGBD camera or onto the MR image when it is registered, shown in Fig. 2b. Secondly, a novel geometric calibration method to register HyperMRI and the external tracking system is applied, enabling the movement HyperMRI, depicted in Fig. 3b.

After the cameras have been calibrated and integrated into the tracking framework, the following essential task is aligning the data captured by all cameras with the MR images. This alignment is critical to create a unified and coherent view of the surgical environment, where all data sources seamlessly complement each other, enhancing the precision of guidance and diagnosis.

Subsequent sections of this paper will provide a detailed explanation of the methodology used to achieve this alignment. This process encompasses aligning HS, RGBD and MR data to ensure smooth coordination between the cameras and the tracking system. This comprehensive approach guarantees that the combined data correspond accurately to the actual surgical scene, benefiting image-guided neurosurgery.

### Calibration methods

#### Homography calibration

The calibration procedure, based on the DLR CalLab framework (German Aerospace Center) [18], is crucial in determining each camera’s intrinsic and extrinsic parameters. Intrinsic parameters encompass perspective projection, lens and sensor distortions, and digitization. Simultaneously, extrinsic parameters are essential for establishing the relative positions and orientations between the infrared (IR) and HS cameras and between the IR and RGB cameras. Radial distortion calibration [24], using the rational function model [4], ensures accurate matching between cameras for proper alignment.

In the calibration process, multiple images of a chessboard are captured from various positions, as depicted in Fig. 2a. It is important to note that all cameras remain stationary while capturing multiple images during calibration. To achieve consistency in calibration, the IR camera serves as the common reference for all three cameras. This strategic choice ensures alignment with depth information, ultimately facilitating precise and accurate calibration.

The obtained parameters are depicted in Fig. 2b. The initial step in the calibration process involves determining the unique intrinsic parameters for each camera. These intrinsic parameters are essential as they serve as the foundation for the subsequent extrinsic calibration procedure. Then, two homography matrices are extracted between (i) IR and RGB cameras and (ii) IR and HS cameras.

**Fig. 4:**
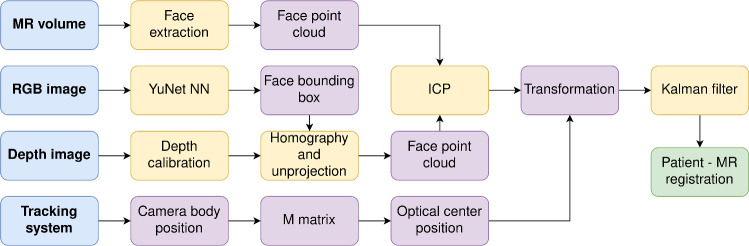
MRI registration overview: a schematic representation of the MR registration process with depth information. Blue represents data sources, yellow denotes algorithms, purple signifies intermediate results, and in green the final registration output

The calibration procedure enables the integration of additional information into each camera. This includes overlaying HS classifications of brain tissues onto the RGB images, a concept previously presented in [14], as well as overlying the HS information onto the MR volume.

#### RGBD—tracking system calibration

Another important calibration procedure in this work involves integrating HyperMRI with a tracking system. The importance of this integration stems from the fact that the patient’s face is visible during the registration step, but as the surgery starts, the face becomes hidden. To address this issue, it is necessary to reposition the camera from the initial view of the patient’s face to the craniotomy area, where the HS camera can capture the exposed brain tissue. To achieve this repositioning, a novel calibration method has been developed to track the cameras’ positions in three-dimensional space accurately. The calibration procedure is shown in Fig. 3.

In this calibration procedure, both HyperMRI and a calibration tool, shown in Fig. 3a, are tracked using Optitrack, an external tracking system [12]. Flat reflective markers are used to ensure the positions of the centre of mass provided by Optitrack and the centre of the marker are identical. To execute this calibration effectively, capturing multiple images using the IR camera of the reference object in different positions is necessary, as shown in Fig. 3a. The primary aim of this calibration is to determine the relative rotation and translation between the centre of mass of the reflective markers in the Optitrack coordinate system and the optical centre of the IR camera (matrix M, in Fig. 3b), as depicted in Fig. 3b.

For each image capture, the positions and rotations of both the camera (C in Fig. 3) and the markers (G in Fig. 3) are determined using Optitrack’s Motive software [12]. When an image is taken with the IR camera, it is necessary to detect the markers within the image. Notably, due to the reflective nature of these markers in the infrared spectrum, the pixel values corresponding to the markers tend to saturate the camera’s sensor. This saturation greatly simplifies their detection in the captured images.

After detecting the markers, the next step involves computing the marker centres to enhance the correlation between the positions provided by Optitrack and those by the camera. Subsequently, correspondences are established between the (x, y, z) coordinates from Optitrack and the (u, v) pixel coordinates from the camera. An efficient correspondence approach has been implemented in this process. The markers on the calibration tool, aligned linearly, are ordered by their x-coordinate inversely to the u-coordinate in the image. In simpler terms, when the camera is focused on the calibration tool, the marker with the highest x-value corresponds to the marker centre with the lowest u-coordinate in the camera image.

Once the markers are detected, and the correspondences are stabilised, a perspective-n-point (PnP) [21] algorithm is employed to estimate the camera pose in the Optitrack coordinate system, obtaining transformation V in Fig. 3b. Finally, the computation of the matrix M is depicted in Eq. 1.

1 $$\begin{aligned} M = C^{-1} \cdot V \end{aligned}$$

### MR registration

The novel registration method introduced in this work relies on the exploitation of the geometric data obtained from the patient’s face to establish alignment with the preoperative MR information. The RGBD camera plays a crucial role in this process. It is responsible for two relevant functions: firstly, it detects the patient’s face in the RGB image, and secondly, it generates the point cloud data of the face by using the depth information, as described in Fig. 4.

A preprocessing procedure is essential to extract the patient’s face from the MR volume information for subsequent use in the alignment process with the depth camera. To accomplish this, it is necessary to align the coordinates of the MR with those of the camera, effectively positioning the patient’s face in the view of a virtual camera. This alignment is achieved by using principal component analysis (PCA) to compute the principal components of the MR volume. The first component, which is perpendicular to the optical axis of the camera, serves as an up vector. The second component aligns with the camera’s optical axis, pointing directly at the tip of the patient’s nose, ensuring precise alignment with the patient’s facial features. With the MR volume aligned to the virtual camera, the next step is to project only the points closest to the camera onto the camera plane using a hidden point removal algorithm [7].

**Fig. 5:**
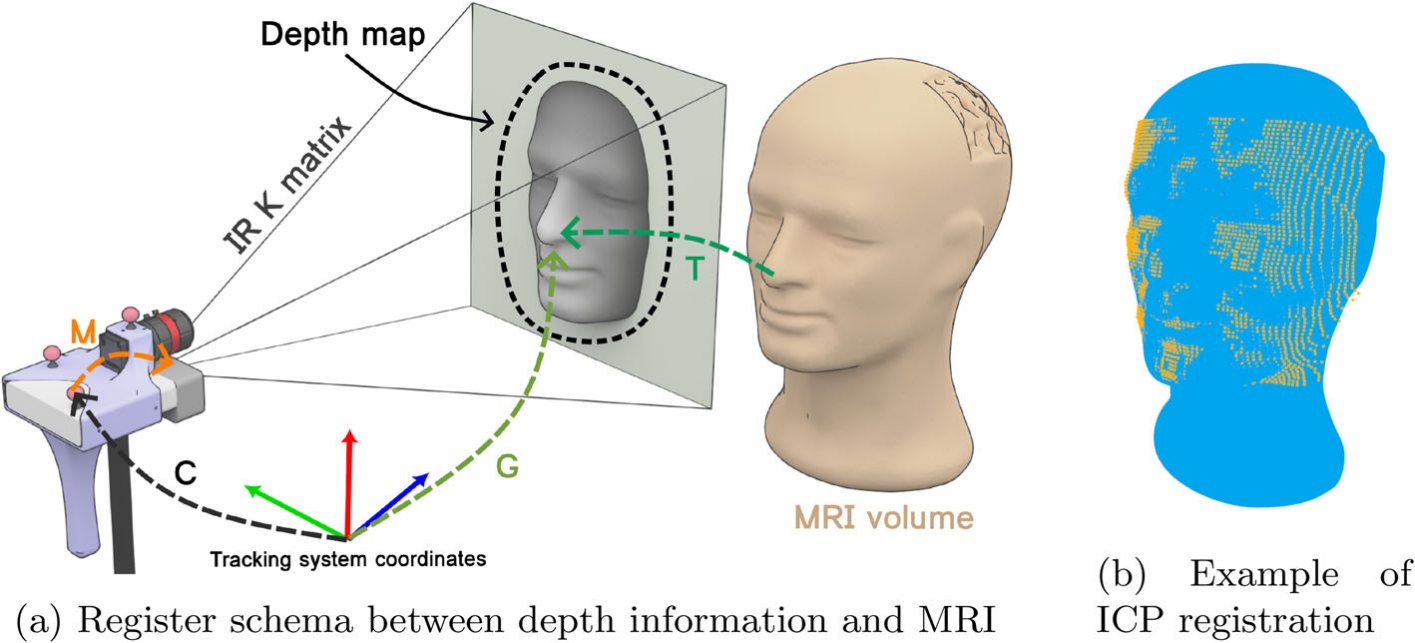
MRI registration. First, the patient’s face point cloud is extracted using depth data from HyperMRI; then, this point cloud is registered with the MR volumetric information using ICP; as a result, a fusion of blue (MR) and orange (face point cloud) data and transform G is obtained

Regarding the RGBD camera, the initial step involves detecting the patient’s face, utilising the YuNet neural network [25]. This neural network provides the bounding box that encloses the patient’s face. Subsequently, the coordinates of this bounding box are projected onto the depth map using the homography matrix that relates the IR and RGB cameras. Only the pixels within the depth map that fall inside this bounding box are then unprojected, this process is facilitated by the intrinsic parameters of the IR camera (K matrix in Fig. 5a). This unprojection creates the patient’s face point cloud while excluding external information that could hinder the registration procedure. Furthermore, a calibration of the depth camera was conducted to minimise measurements errors characterised by the manufacturer [10]. Using the tracking system, the distance between a white plane and the camera was measured, compared to the depth provided by the camera at intervals of 5 cm along an 80 cm range, repeated 25 times. These data were fitted to a curve to correct the depth of the patient’s face.

**Fig. 6:**
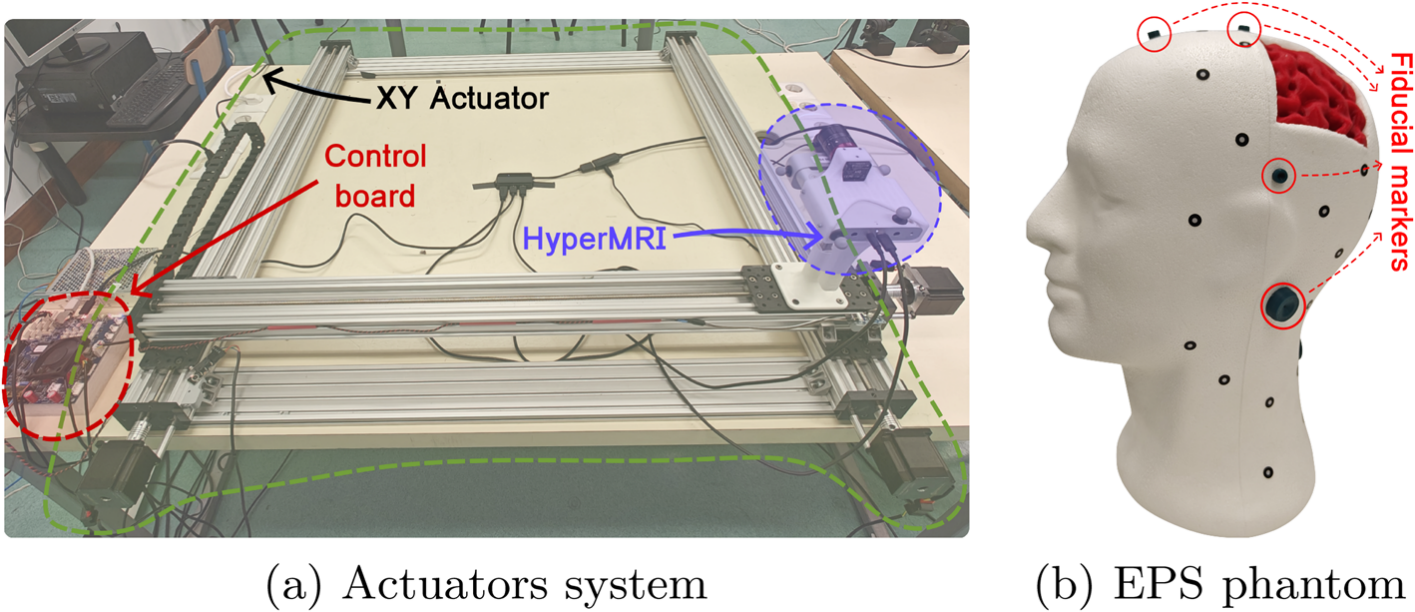
Experiment materials

Once the point cloud of the patient is extracted, it is provided to the iterative closest point (ICP) algorithm [17] along with the patient’s face data from the MR. To enhance the registration process, an initial transformation is computed using the RANSAC method [3], which serves as the preliminary step for the ICP algorithm until convergence. This resultant transformation (T in Fig. 5a) represents the relationship between the MR and the RGBD camera in that specific position. This transformation is further adjusted to enable camera movement by incorporating the current camera position (C in Fig. 5a) following Eq. 2 to obtain the absolute position of the MR in Optitrack’s coordinate system (M in Fig. 5a). This process is repeated along the time, computing a new transformation matrix by frame and feeding a Kalman filter with these transformations to mitigate the noise in the depth map and the tracking system and obtaining a final alignment between the MRI (blue point cloud) and the actual face of the patient (orange point cloud) as depicted in Fig. 5b.

2 $$\begin{aligned} G = C \cdot M \cdot T^{-1} \end{aligned}$$

## Experimental results

The feasibility of the methodology and system was evaluated using a human head phantom (Fig. 6). Following the approach in [5], a patient’s complete head was not recreated; instead, an expanded polystyrene (EPS) head served as the base. A brain segment was 3D-printed based on a real MR image and integrated into the EPS head to visually assess the alignment between MR and HS data. The MRI data were obtained from the 3D-printed head using an EinScan Pro 2X 3D scanner (Shining 3D) and quantified using 3D-Slicer software to create the MRI volume.

For consistent registration experiments, a 2-degree of freedom system, as illustrated in Fig. 6a, was constructed. This system comprises an XY linear actuators that facilitate camera movement within a plane parallel to the ground, assembled using aluminium rail slots and motors. The system is controlled with a Duet 3 Main Board 6HC and RepRapFirmware 3 firmware achieving 6400 steps/mm resolution.

To establish a baseline reference in the registration process, a fiducial marker approach was employed, using a Micron Series Digitizing probe (Optitrack) in conjunction with 3D-Slicer and the SlicerIGT extension [23].

### Homography calibration results

As previously mentioned, a critical step in the methodology proposed in this work involves the intrinsic and extrinsic calibration of the different cameras comprising HyperMRI. The calibration process, as described in “Homography calibration” section, entailed capturing 8 images for each camera using a radon chessboard with a grid of $32\times 25$ corners. To enhance the quality of the obtained parameters, all captures were interpolated to a $3840\times 2160$ pixels resolution, except for the RGB camera, which already features that resolution (IR native resolution $640\times 576$, HS native resolution $409\times 217$). To assess the calibration’s quality, a reprojection root-mean-error (RMSE) in pixels was retrieved for each camera by the CalLab software. Also, this error is computed for each pair of cameras, measuring the quality of the extrinsic parameters. The results obtained are presented in Table 1 using the interpolated 4K resolution in each camera.

**Table 1:** Reprojection RMSE [pixel] for each camera and between them in 4K resolution

	IR camera	RGB camera	HS camera
Intrinsics	0.67	0.74	16.22
Extrinsics	Ref.	0.71	5.17

**Fig. 7:**
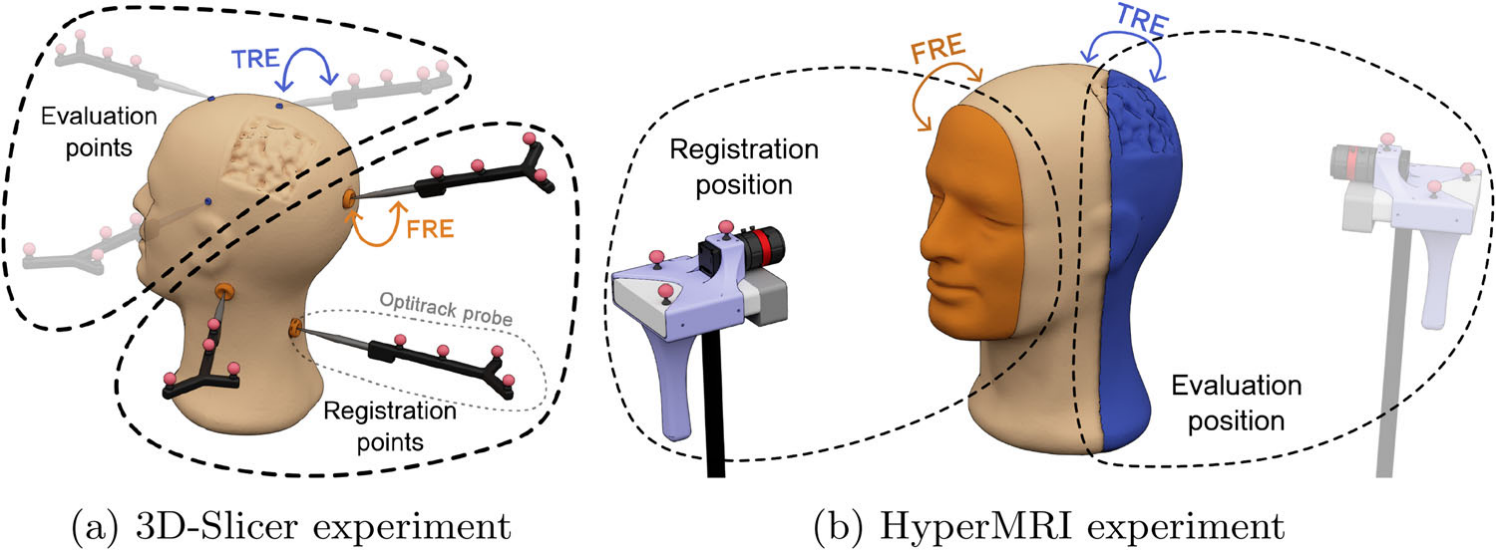
Experiment schema for 3D-Slicer and HyperMRI. Orange elements represent landmarks and point clouds used for the registration procedure to extract the FRE metric, while blue elements are used to compute the TRE metric

### RGBD—Tracking system calibration results

In the context of the calibration of the optical centre of the IR camera with the tracking system, it is important to note that the Optitrack system has its own calibration procedure to ensure proper functionality. The Optitrack system was calibrated using the manufacturer’s tools, resulting in a reprojection RMSE of 0.34 ± 0.1 mm in an approximate volume space of $4\times 3\times 2$ m.

Concerning the novel calibration method described in Section “RGBD—Tracking system calibration", 20 captures of the IR camera were utilised, with the corresponding coordinates of the reference object incorporated. To gauge the accuracy of this calibration, the reprojection RMSE was employed, resulting in an error of 1.92 ± 0.54 pixels.

### MR registration results

To evaluate the quality of the MR registration methodology a pilot study making use of the phantom, probe, actuator system and reference software described at section “Experimental results” was conducted. The experiments concern two quality evaluations: one assesses the performance at the registration position, measuring the Fiducial Registration Error (FRE), and the other uses points not involved in the transformation computation, quantifying the Target Registration Error (TRE). Both the HyperMRI system and the 3D-Slicer system have been characterised in this way, as illustrated in Fig. 7. In these experiments, the automatic processes were repeated 25 times, while the manual ones were repeated 5 times to extract the mean and standard deviation of the data.

The characterisation procedure of the baseline reference using 3D-Slicer involves two distinct steps, illustrated in Fig. 7a: (i) the registration, which entails selecting landmarks in the preoperative image using 3D-Slicer and establishing their correspondence by using the Optitrack probe, and (ii) the evaluation, where the same process is repeated with points not used in the registration to compute the distance between them and the MR points transformed with the registration transformation. The outcome of the first step is the FRE metric, while the outcome of the second is the TRE. These results are introduced in Table 2, where each experiment is repeated 5 times.

Concerning the HyperMRI system, two experiments were conducted, mirroring the methodology used in the 3D-Slicer experiments but employing the point cloud retrieved by the depth camera instead of sample points, as shown in Fig. 7b. For the registration procedure, a total of 25 positions were examined using the actuator system, arranged in a $5\times 5$ array with a 10-cm gap between each position. The FRE was computed by determining the distance between the point cloud extracted from the patient’s face (orange surface in Fig. 7b) during the registration process and the corresponding region in the preoperative image. For the evaluation procedure, 5 registrations were performed to extract the TRE metric. The camera was subsequently moved to the evaluation position, where all visible points on the phantom head (blue surface in Fig. 7b) were utilised to compute the TRE metric making use of ICP to avoid human bias. The results of both metrics are presented in Table 2.

**Table 2:** Registration errors and times for HyperMRI and 3D-Slicer

Method	FRE (mm)	TRE (mm)	RRE (mm)	Time/register
3D-Slicer	1.1 ± 0.25	3.11 ± 0.80	Ref	< 4 min
HyperMRI	1.88 ± 0.19	4.07 ± 1.28	3.98 ± 1.02	5.25 ± 0.68 s

**Fig. 8:**
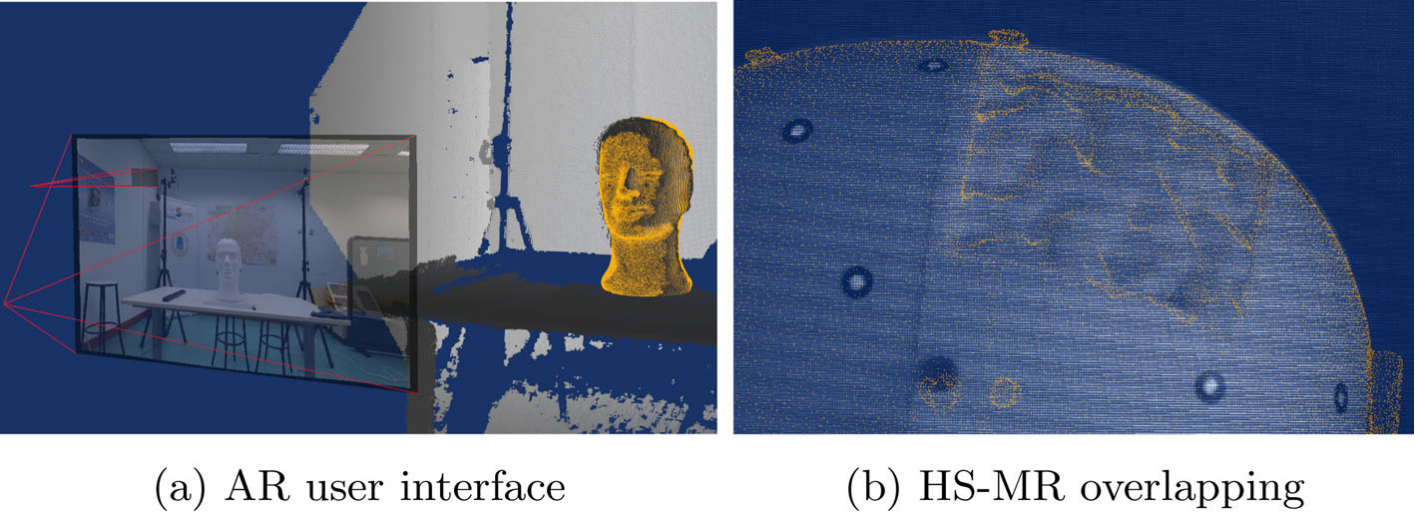
AR user interface. **a** MR (orange) registered with the depth information (grey), and the RGB and HS image planes. **b** MR (orange) HS image overlapping

Finally, a comparative analysis was conducted using the 25 registered positions used for HyperMRI’s FRE characterisation. The distances between landmarks in the MR images transformed with 3D-Slicer and with HyperMRI registration were computed and referred as Relative Registration Error (RRE) in Table 2.

### AR user interface

To visualise the overlap between the HS image and the MR information, an augmented reality user interface was developed. This interface was created using Python and leveraged the VTK library for HyperMRI data representation.

As illustrated in Fig. 8a, the point cloud extracted from the depth camera is displayed in greyscale, the MR volume is represented in blue, and the RGB and HS images are shown on planes. The user interface allows to fix the point of view on one of the cameras, e.g. the HS camera, as demonstrated in Fig. 8b (note the overlap between MR and HS images in greyscale).

The position of HyperMRI is updated in real time based on the information provided by the Optitrack system. Additionally, the point cloud and images are updated at a smooth frame rate of 30 frames per second. This user interface enables to visualise the overlap between the HS and MR information, as shown in Fig. 8b, and also to easily navigate through the MR volume and the 3D environment.

## Conclusion and future work

This paper has introduced the HyperMRI prototype and a comprehensive methodology for precise patient registration in neurosurgery applications. The proposed methodology seamlessly integrates additional information, such as HS technology and RGB images, into the patient’s preoperative MRI within a AR environment. Moreover, the incorporation of a novel calibration method with a tracking system enhances the potential of the HyperMRI system making possible a seamless integration into an actual neuronavigation environment. This cost-effective and user-friendly design, as an evolving prototype, holds the potential to further introduce HS in the image-guided neurosurgery field by means of the widely validated technology of MRI. The integration of HS technology with MR information in the computer-assisted surgery (CAS) environment could, for example, improve tumour border determination through tissue classification and enable the use of HS-detected blood vessels for brain shift correction.

The system demonstrates a satisfactory level of accuracy in the registration task, with the primary limitation being the depth quality of the Azure Kinect DK camera. This camera exhibits a systematic error of approximately 11 mm and a random error standard deviation of around 17 mm, as reported in [10], which introduces minor differences into the registration procedure. These errors have been mitigated by the proposed methodology to improve the registration accuracy. The system achieves a FRE of 1.88 mm and a TRE of 4.07 mm in an automatic procedure without human bias, completing the process in 5 s. In contrast, manual landmark registration achieves a slightly superior quality (3.11 mm TRE) with the counterpart of being dependent on the operator’s experience and requiring several minutes for the registration. To further enhance accuracy and reduce error, future iterations of this prototype may benefit from a more metric-accurate depth camera with higher resolution or specifically designed for this use case (for example with a closer working distance). All the code and the data used in this work are public available.^1^

In the near future, it is awaited to evaluate the performance of HyperMRI in real clinical environments to assess the system’s quality under practical conditions. Additionally, it is expected to integrate the tracking system directly into the HyperMRI system. This integration will involve only using external markers to estimate the camera’s position both in the registration area and within the craniotomy site, and not the Optitrack system. This approach aims to reduce the equipment needed for system integration in an operating room, reducing it to just two cameras and simplifying the overall incorporation process.

## Footnotes

1 https://gitlab.citsem.upm.es/public-projects/immersive-imaging/hypermri-system.
